# Triggering multiple sclerosis at conception and early gestation: The variation in ultraviolet radiation is as important as its intensity

**DOI:** 10.1016/j.heliyon.2023.e16954

**Published:** 2023-06-03

**Authors:** George E. Davis, Matthew J. Davis, Walter E. Lowell

**Affiliations:** Riverview Psychiatric Center, 250 Arsenal Street, State House Station #11, Augusta, ME, 04333-0011, USA

**Keywords:** Multiple sclerosis, Month-of-conception, Month-of-birth, Ultraviolet radiation, Latitude, Elevation, Coefficient of variation, Sunspot number

## Abstract

**Background and objectives:**

Medical science needs to further elucidate the role of ultraviolet radiation (UVR), geographic latitude, and the role of vitamin D in the autoimmune disease multiple sclerosis (MS). We separated several papers into categories out of the thousands published and used their conclusions to explore the relationship between UVR and MS.

**Relevance:**

MS is increasing in incidence, particularly in women where MS is two to three times that in men and particularly severe in African Americans.

**Methods:**

We collected UVR data at our observatory in Central Maine and calculated the average coefficient of variation (CV_UVR_) for each month for 15 years (2007–2021, inclusive).

**Results:**

The month of conception (MOC) is more important than the month of birth (MOB) in explaining how UVR triggers the variable genetic predisposition to MS. We hypothesize that the rapidly increasing CV_UVR_ is important in preventing an increase in the activity of the vitamin D receptor (VDR) from August to December, which then requires a higher intensity of UVR later in life to suppress the immune system, therefore predisposing to more MS.

**Limitations:**

One observatory at about 44° latitude.

**Conclusions:**

While variation in UVR is important at the MOC if UVR exceeds a threshold (e.g., if the sunspot number equals or is greater than 90, usually at a solar cycle MAX, or at elevations above approximately 3,000 feet above sea level), the MS mitigating vitamin D-VDR mechanism is overwhelmed and the genotoxic effects of higher-intensity UVR promote MS in those with a genetic predisposition.

**What is new in this research:**

This paper offers a new concept in MS research.

## 1 Background & introduction

Multiple sclerosis (MS) is an inflammatory, demyelinating, and neurodegenerative autoimmune disease of the central nervous system (CNS) and spinal cord that expresses non-Mendelian genetics and affects approximately 2.8 million people worldwide [1]. The country with the largest incidence of MS is Canada (290 cases/100K as of 2011), while northern European countries, especially Denmark, Sweden, and the Faroe Islands also have a high incidence. The disease is at least two to three times as common in women with the highest prevalence age being 45–49 years [2]. The prevalence of MS in the United States is estimated to be 309/100K (450/100K for women and 160/100K for men) a female/male ratio of 2.8 [3]. In the USA southern states (below 37° latitude) the incidence of MS is between 57 and 78 cases/100K population. The rate is twice as high in the northern states (above 37° latitude) to about 110 to 140 cases/100K population.

During the period of data collection for this study 2007–2021, inclusive, e.g., 15 years, a PubMed search using only “multiple sclerosis” in the title yielded 29,086 scientific papers concerning the diagnosis, pathogenesis, and treatment of MS. Many of these articles concerned treatment but etiology and pathogenesis remain challenging. We grouped some of the more recent papers into broad categories to help the reader appreciate the complexity and sometimes contradictory findings as a background for understanding the pathogenesis of this uncommon, but not rare, disabling disease. Conclusions or paraphrasing of the conclusions in these papers follow.

### 1.1 Latitude versus ultraviolet radiation (UVR)

We, [GED & WEL], reported that in the United States, there is a linear relationship of the percent of MS patients to latitude based on a population centroid of cases [4]. The frequency of MS approached zero at the Tropic of Cancer (approximately 23.5° N) supporting the effect that UV-B is at least part of the pathogenesis of MS. A similar finding by other authors found that MS incidence is greater above 40° N (or S) latitude [5]. A latitudinal prevalence gradient also exists in Latin America between Panama and Argentina [6]. However, the latitude gradient did not fit an Italian map for MS, especially in the islands of Sardinia and Sicily [7]. Living in a high UV-B environment with summer exposure during childhood in the years leading up to MS onset was associated with a lower MS risk [8]. However, a Japanese study reported that latitude has more of an impact on the prevalence of MS than UVR intensity or duration [9]. MS risk increased as average lifetime levels of UVR exposures in winter decreased, especially across age groups less than 40 years of age. There was little indication that low UVR exposure during summer or in old age was related to MS risk. The findings are consistent with the hypothesis that UVR exposure reduces risk [10]. UVR has also been associated with a delayed relapse rate for MS [11,12]. A negative correlation of MS incidence with UV-B is stronger than with latitude in one study with the correlation strongest in the first year of life and declining in years up to age 10 that suggests UV-B contributes to the pathogenesis of MS [13]. In a study of 11,415 patients from 15 countries, the seasonal fluctuation of MS births did not correlate with variation in the seasonal fluctuation of UVR [14]. Latitude also appears to be more important than levels of vitamin D as a study in the Canary Islands (29° latitude) and peninsular Spain (36° latitude) showed no difference in vitamin D levels between MS patients and healthy controls [15,16]. A new meta-analysis confirms that MS prevalence is positively associated with increasing latitude and the MS gradient is increasing, suggesting that potentially modifiable environmental factors such as sun exposure, are strongly associated with MS risk [17]. In China there is a north-south latitude gradient, and in addition, a west-east altitude gradient (with more MS at higher elevations) [18]. Significant variation in the incidence of MS has occurred in the Faroes over time suggesting that environmental factors are more important than genetic factors [19]. A Polish study of 2,574 patients with MS revealed a seasonal variation in the risk of MS, but this finding did not correlate with hours of sunshine (e.g., UVR dose) during the first trimester of pregnancy [20].

In comparing North American regions with the United States in a case-controlled study of MS patients, the risk ratio (RR) of the lowest and highest UV index (UVI), was 3.78, and within the U. S. regions 1.52, supporting an increased MS risk in those areas with a low UVI [21].

An earlier age-at-onset of MS in higher latitude regions was found in this world-wide European-descent cohort and correlated inversely with variation in latitudinal UVR [22]. Not all research agrees with the effects of UVR on MS and some have postulated increased temperature as a factor [23].

### 1.2 Ultraviolet radiation, vitamin D, the vitamin D receptor, and the immune system

UVR, particularly the 5–6% moiety UV-B (280–315 nm), produces vitamin D, a hormone involved with calcium absorption and immune modulation. The vitamin D receptor (VDR), in virtually all our cells, acts as a transcription factor and regulates several vitamin D-responsive genes, including those involved in the immune system [24]. The VDR executes most of the biological functions of vitamin D [25]. The VDR binds to thousands of genomic loci and modulates, through local chromatin changes, the expression of hundreds of primary target genes. The epigenome and transcriptome of VDR-expressing cells is directly affected by vitamin D [26]. Recent papers have linked vitamin D deficiency to inflammation, and probably by extension, increased autoimmunity, which would include MS. In that paper vitamin D promotes the production of IL-10, an anti-inflammatory cytokine and markedly decreases C-reactive protein (CRP) in cases with the most severe vitamin D deficiency [27]. The production of vitamin D may also serve to offset the genotoxic/mutagenic effects of UVR, and in this respect, moderate sun exposure is salutary. Vitamin D supplementation modulates seasonal MS activity, especially during the late winter and early spring [28].

### 1.3 The month of birth and incidence of multiple sclerosis

There is an increased incidence of MS in the peak month of April compared to the trough month of November (15.7% fewer) [29]. In the Norwegian population born between 1930 and 1979 (N = 2,899,260), there appears to be a persistent higher-than-expected frequency of April births in the MS population [30]. Another study found that more persons with MS were born in the month of May (N = 42,045 pooled patients from several countries) [31]. The MOB effect was less prominent in geographic regions with high UVR exposure but more evident in areas with low UVR exposure [32]. For births in April and May, conception would occur in July and August, respectively. The significance of this observation will be discussed subsequently.

### 1.4 MS related to other diseases

Our research group recently reported that UVR intensity and variation promoted major mental illness (MMI); e.g., schizophrenia (SZ), bipolar disorder (BPD), and schizoaffective disorder (SZAFF) [33]. We found that more patients with SZ were born in March, also in December. Born in March means conception in June with maximum solar light at the summer solstice; likewise, being born in December means conception in March at the spring equinox, a time of rapid change in solar irradiance. It is also known that patients with MS appear to have an increased risk of developing schizophrenia or bipolar disorder suggesting that UVR may also be a trigger for these diseases [34]. Seasonal UVR appears to modulate the expression of other CNS diseases the most common being major depression [35].

### 1.5 The link between the vitamin D, multiple sclerosis, and pregnancy

Vitamin D acts as an immune regulator during implantation providing a protective effect during the entire pregnancy [36]. The presence of vitamin D deficiency was 96% in Ethiopian MS patients demonstrating that even living 9° above the Equator with high UVR irradiance, dense cutaneous melanin along with protective clothing, inhibited adequate vitamin D production [37]. Women with recurrent pregnancy loss (RPL) have lower VDR placental and serum expression compared to normal pregnant women; decreased VDR expression in the first trimester of pregnancy is likely associated with RPL indicating an abnormal immune mechanism [38]. Women with increased VDR expression in the endometrium, especially during the implantation window of the menstrual cycle, were significantly more likely to be pregnant than women with decreased expression. The results support the hypothesis that the vitamin D-VDR system performs a role during the development of endometrial receptivity [39].

The latitude gradient for MS, largely driven by females with relapse onset MS, is established in early life *in utero* with the slope of the gradient persisting until age 12 before gradually declining [40]. In an Australian study low maternal exposure to UVR in the first trimester of pregnancy was associated with a subsequent risk of MS in offspring [41].

### 1.6 Virus infections and the incidence of MS

The latitude gradient in the prevalence of MS in France is not uniform depending upon the socioeconomic status of the population. The discrepancy of distribution between farmers and independent workers on the one hand and employees on the other cannot be attributable to environment. This favors possible viral infection as a contributory etiology for MS [42].

The prevalence of MS in Telemark, Norway, among the highest ever reported in Norway, is consistent with an increasing incidence in the country over the past twenty years. The even higher prevalence in rural areas is unlikely to be explained by possible risk factors like latitude, exposure to sunlight, and diet [43]. This again increases the probability of a viral etiology like Epstein-Barr (EB) virus. The risk for MS was 32-fold higher after EB infection, but not increased with other viruses. EBV may be a leading cause for MS [44].

### 1.7 Negative relationships between MOB and MS incidence

In 12,020 Swedish and 108 Icelandic MS patients no significant difference was seen between expected and observed MS births related to season or MOB [45]. After correcting for MOB patterns in the general population, there is no evidence for the previously described MOB effect in Austrian MS patients [46].

### 1.8 Psychiatric manifestations in MS

Seasonal affective disorder (SAD) was described in 1984 and was the first psychiatric disorder to be reported to be affected by rapidly decreasing light in autumn [47]. Psychiatric symptoms may be the first clinical presentation of MS [48]. Most patients (56%) presented with a mood disorder with characteristics of a major depressive-like episode with five (32%) with psychotic symptoms. Psychiatric symptoms at onset of MS may be indicators of possible maintenance of psychiatric morbidity in a sizeable proportion of patients and occasionally psychiatric symptoms may be the only manifestation of MS [49]. MS can manifest with myriad psychiatric symptoms, with studies showing between 60 and 95% of MS patients showing evidence of some type of psychiatric symptom [[50], [51], [52]]. Most prominent are affective (mood) disorders, specifically major depressive disorder, where the lifetime prevalence in MS patients approaches 50% and where more than a quarter of patients have suicidal ideation at some point during the course of illness [50,53]. There is considerable overlap between MS symptoms and neurovegetative symptoms of a major depressive episode, specifically, sleep disruption, energy changes/fatigue, and cognitive deficits such as impaired concentration. This may make differentiating a major depressive episode from an initial presentation of MS or an MS flare particularly challenging for clinicians. At present, it is unknown whether the pathophysiological changes associated with MS induce major depression or whether major depression is a consequence of psychosocial stress secondary to a lifelong, debilitating illness [54] Interestingly, major depression occurs more commonly in MS patients than in patients with other chronic, debilitating neurological conditions suggesting that the disease process itself plays a direct role in inducing major depression [54]. In a Canadian study of 495,739 patients with major depressive disorder, there was a 1–2% increase in the prevalence per degree of latitude indicating that another mental illness besides SZ, BPD, or SZAFF disorder is affected by latitude, and by extension, sunlight [35]. A majority of MS patients do not receive adequate treatment for major depression even though it is often treatable with psychopharmacology and/or psychotherapy [55]. Bipolar illnesses are rarely associated with MS. Manic episodes may manifest prior to the onset of neurological symptoms, secondary to an MS flare, or due to a drug-induced etiology (in particular, corticosteroids, which are known to induce manic symptoms generally). Typical treatments, such as lithium and neuroleptic agents, are often helpful in treating mania associated with MS [56]. Our own research in the USA reported that latitude is directly related to *variation* in suicide rates suggesting that the pattern of *variation* in sunlight (probably UVR) is superimposed on human mood by some mechanism [4]. A large change in solar irradiance between winter and summer and between the minimum and maximum monthly values may increase the risk of suicide attempts in BPD (N = 1,496) where the odds of a suicide attempt decrease by 4.4% for every 0.1 increase in the ratio of mean winter to summer insolation [57].

### 1.9 Racial differences in the incidence of MS

African American patients have more severe disease [58]. The percentage of females with MS was higher in African Americans than in Caucasians (83% vs 47%, respectively) suggesting a more aggressive disease phenotype in African Americans with MS. African Americans have a greater risk for disability if they contract the disease [59]. There is more transverse myelitis and optic nerve involvement in African Americans with MS (16.8%) versus Caucasian Americans (7.9%) indicating more aggressive disease in the former [60]. There is an increasing incidence of MS in African Americans but Hispanics and Asians are less at risk compared to Caucasians [61]. People with a darker skin tone have lower vitamin D levels and an increased risk of MS, but this does not explain why Hispanics and Asians have a lower risk for MS than Whites or why the higher risk of MS among African Americans was found only among women [62]. Racial/ethnic variation in bioavailable vitamin D does not explain the lack of association between 25-hydroxyvitamin D and MS in African Americans and Hispanics; this challenges the plausibility of vitamin D deficiency as causal for MS [63]. There was no apparent association between vitamin D status and MS disease severity even though the levels of 25-hydroxyvitamin D were lower in African Americans with MS than controls, explained by differences in climate and geography [64].

### 1.10 Genome versus epigenome in the expression of MS

In the first systemic effort to estimate sequence variation among monozygotic twins, there was no evidence for genetic, epigenetic, or transcriptome differences that explain MS disease discordance. The study is the first female, twin, and autoimmune disease individual genome sequences reported [65]. Various common MS-associated risk SNPs (single nucleotide polymorphisms) are located within the vicinity of genes associated with the complex metabolism of vitamin D. The association between vitamin D and MS could be based on the extensive and characteristic genomic binding of the VDR [66]. Proband-wise concordance rates of 25.3% ( ± SE 4%) in monozygotic twins; 5.4% in dizygotic twins [67]. This monozygotic rate is close to the theoretical predicted 28% rate indicating an environmental; e.g., the epigenetic moiety that cannot be eliminated even in genetically identical people [33]. Consanguinity does not appear to be an important risk factor for MS in the genetically relatively homogeneous population of the Faroe Islands [68].

### 1.11 Effects of UVR on puberty

There is an inverse association between age at menarche and MS risk. For each year increase in age at menarche, the risk of MS was reduced by 13%. Early age at menarche appears to be associated with an increased risk of MS [69]. Women born at lower latitudes or in regions with higher annual UVR dose had a 3- to 4-month earlier menarche than women born at higher latitudes or in regions with lower UVR [70].

## 2 The DATA

We [GED] collected ground-based UVR data at our observatory for 15 years from year 2007 through 2021, inclusive, encompassing Solar Cycle 24 (December 2008 through December 2019). Table 1 lists the coefficient of variation (CV) defined as the standard deviation divided by the mean UVR in MEDs (MED is defined as 210 J/m^2^) The CV_UVR_ is a unitless metric so that one may conveniently compare data with those using other units of measurement. Latitude and longitude of the collection site are listed in the title of Table 1. Details of data collection are outline in the Methods section.

**Table 1:**
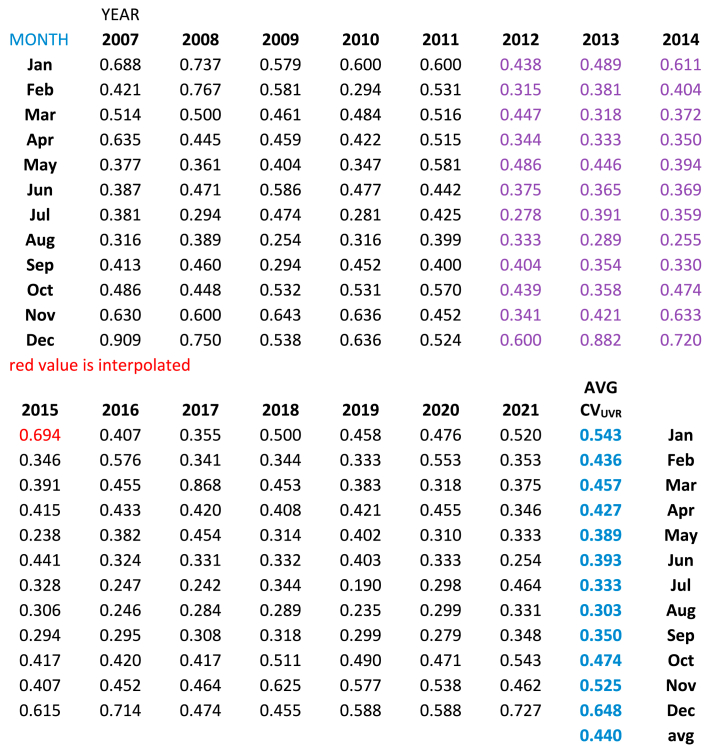
Coefficient of variation of UVR from years 2007–2021, inclusive at Augusta, Maine, latitude 44.309, longitude −69.769, elevation 124 feet.

## 3 Methods

### 3.1 Instruments and definitions

We used a UVR pyranometer (Davis Instruments Corp. 3465 Diablo Ave. Hayward, California 94545 USA) with a spectral response of 280 to 360 nm (Erythema Action Spectrum) with an accuracy of ± 5% of daily total dose recorded as MED (Minimal Erythemal Dose) for a medium-pigmented skin where 1 MED = 250 J/m^2^). UVR wavelength definitions: UV-C (100–280 nm), UV-B (280–315 nm) which causes cyclo-pyrimidine dimers in the epidermis, and UV-A (315–400 nm) which causes oxidative damage and DNA base modifications through reactive oxygen species (ROS) in the dermis. A data logger recorded UVR every one-half hour during daylight hours. If one estimates 12 h of daylight/day for 365 days, that would be 24 data points x 365 days × 15 years = 131,400 data points, minus 720 lost data (Jan 2015, subsequently interpolated) = 130,680 data points collected for this study. Of note, UVR comprises about 94–96% UV-A and 4–6% UV-B depending upon the season. The lack of high pyranometer accuracy ( ± 5%) was not an issue as we were interested in each month of the year averaged over 15 years and any deviations in accuracy would be shared among all the months. Our main interest concerned the *relative changes* between months. The units of UVR are in MEDs at 250 J/m^2^, recently changed to 1 MED = 210 J, but a more modern unit is now the SED (Standard Erythema Dose) which by convention is equal to 100 J/m^2^.

Note that we calculate the month of conception (MOC) as 10 months (40 weeks) before birth because gestation is calculated from the first day of the last menstrual period or 280 days, but if one includes late-term and post-term pregnancies, the gestation period can extend to 42 weeks or 294 days.

Other instruments used in this study were two pyranometers: Solarmeter Model 5.0 for total UV-A + UVB 0–199.9 mW/cm^2^ ( ± 10% accuracy) in the 280–322 nm range and Solarmeter Model 6.2 0–1999 μW/cm^2^ ( ± 5% accuracy) in the 280–400 nm range. Both instruments are produced by the Solar Light Company, LLC 100 E. Glenside Ave., Glenside, PA 19038, USA. Using these instruments around the autumnal equinox at 44° latitude, −70° longitude (in Maine), the UVR irradiance at ground level of UV-A + UV-B = approximately 30 W/m^2^ of which UV-B alone is measured 1.2 W/m^2^, or about 4% of the total UVR. The reported ratio UV-B/(UV-A + UV-B) at the summer solstice at our site is 6.3%; at the winter solstice 3.3%. The irradiance at ground level averages 56 W/m^2^ at the summer solstice. Through a double-paned, gas-insulated window glass: UV-A + UVB = 1 mW/cm^2^ (equals 10W/m^2^); UVB alone = 4 μW/cm^2^ (0.04W/m^2^); the ratio UVB/UV-A + UVB = 0.004 or 0.4%; that is, most UVB is blocked, but 1/3rd 10/(30*0.96) = 0.34) of UV-A does penetrate.

In previous research we found that sunspot number (SSN), a surrogate for solar intensity, greater than or equal to 90 (the average SSN is about 50 over several solar cycles) was associated with an average 8-year shortened lifespan, implying genotoxicity [71].

Note that UVR rapidly changes at both equinoxes, e.g., a rate of change. However, variation in UVR, e.g., CV_UVR_, relates to standard deviation and increases from summer to winter.

**Fig. 1:**
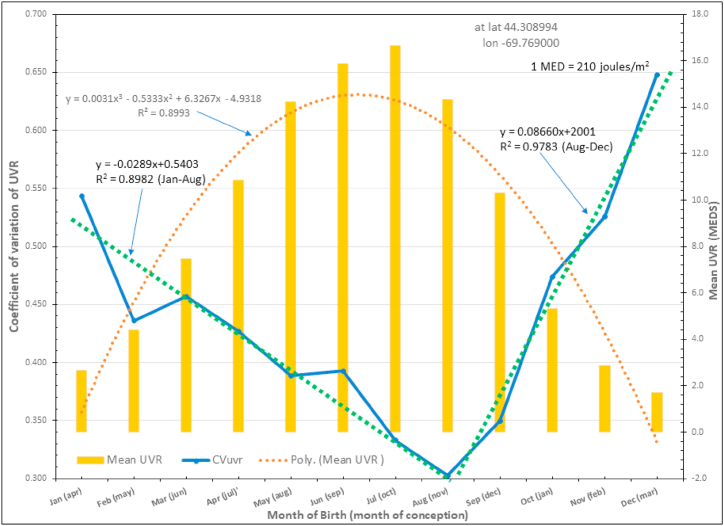
Mean Coefficient of Variation of UVR and mean UVR dose by month over a 15-year period from 2007 to 2021, inclusive.

For this paper we averaged the CV_UVR_ for each month as seen in Table 1 and plotted these averages as seen in Fig. 1. We applied trend lines to the January–August and to the August–December portions which were virtually linear, especially the latter portion.

## 4 Results

Referring to Table 1, the column labeled “AVG CV_UVR_”, colored blue and in bold type, is plotted in Fig. 1. Note that the horizonal axis is labeled MOB, but in parentheses are the corresponding MOC occurring, as we defined, ten months before birth. When the CV_UVR_ is plotted there are two effectively linear lines meeting at a 90-degree angle with the nadir at the month of August with the lowest value of CV_UVR_ = 0.3. The positive slope of the line from August to December is exactly three times (0.087/0.293 = 3.0) that of the negative slope of the line drawn from January to August. Also, in Fig. 1 the secondary Y-axis is the mean UVR in MEDs by month, each averaged over the 15 years. The lowest CV_UVR_ does not occur at the summer solstice around June 21st, but instead in August at our location in Maine. The average CV_UVR_ is 0.440 which is the value at both equinoxes. For the three MAX years 2012, 2013, and 2014 in Cycle 24 the average CV_UVR_ is 0.391, 13% less than the overall 15-year average (see Table 2).

**Table 2:**
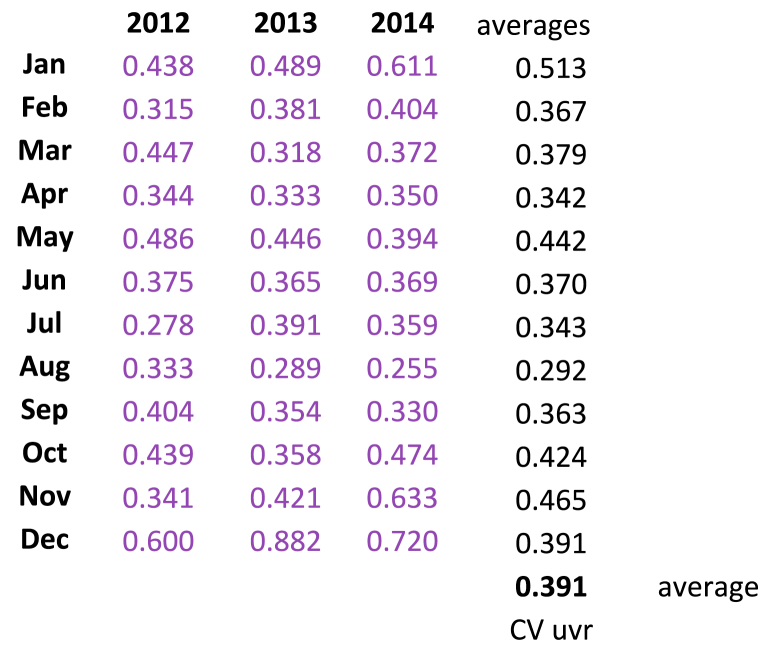
Average CV_UVR_ for MAX years of Solar Cycle 24.

According to the above references, and referring to Fig. 1, the most common MOB for MS occurs in April or May, the least common MOB for MS is in November [29].

For May, the MOC would be in August and, as seen in Fig. 1, UVR has the lowest variation of 0.30 in a decreasing, but still high, irradiance of UVR. As in the example of exogenous opioids suppressing mu pain receptors, strong UVR suppresses the nominal activity of the VDR or other UVR-sensitive embryonic receptors or chromophores (through maternal integument, to be discussed subsequently). During the first and part of the second trimester of pregnancy (September through December) decreasing UVR that would logically increase VDR is thwarted by a rapidly increasing variation in UVR at a rate three times that of decreasing CV_UVR_ from January to August. We hypothesize that this UVR dynamic, dispersing UVR like a shotgun, suppresses VDR receptors overriding the decreasing UVR irradiance of late fall and early winter. This supports the findings of Staples et al. who found that low UVR exposure in the first trimester of pregnancy was associated with more MS in progeny [41]. The requirement for UVR to produce vitamin D and to suppress the immune system would be greater with fewer VDRs fixed later in pregnancy.

If one were to use an example of a person with genetic loading for MS who was born in August, the MOC being November, VDR receptors would start with a nominal activity likely at the autumnal equinox, however, further development in the first trimester (December, January, and February) would occur in relatively low UVR irradiation with a slowly decreasing CV_UVR_ which would favor more VDR receptors. We would predict an MS patient born in August to have milder disease relative to April or May births which comprise the 30% of the more serious MS courses, including primary progressive MS [[72], [73], [74]].

Taking November as the MOB, MOC being February, the UVR in the first trimester of March, April and May is increasing, but not maximal, while at the same time CV_UVR_ is decreasing, both dynamics bring the VDR to a nominal activity. Receptors of UVR are sufficient to adequately bind to vitamin D, and through UV-A, suppress an overactive immune system resulting in the lowest incidence of MS and the least activity of the disease. The average CV_UVR_ of the MAX years of Solar Cycle 24 is 0.391 (see Table 2), which is 13% lower than the overall average. Less variation means more potential suppression of VDR receptors and an increased requirement of UVR exposure later in life to mitigate MS activity. Moreover, the increased genotoxic/mutagenic effects of UVR are not salutary as persons living at higher elevations have a greater incidence of MS despite increased production of vitamin D [18]. Note that a SSN greater or equal to 90, can also occur during solar MIN (about 8 years) although less often than during MAX (about 3 years).

## 5 Discussion

This purpose of this paper is to offer a new hypothesis as to the etiology of MS and to explain some of the contradictions in recent, extensive published literature. The concept of early-life events having a profound influence on the phenotypic expression of adult disease is due to the efforts of pioneers like A. Vaiserman, M. Lucock, P. Gluckman, among several others [[75], [76], [77], [78], [79]]. Early-life exposure to light may affect the long term adaptability to respond to circadian challenges in later life [78]. The cause of some of the confusion regarding MS and the relationship to MOB, latitude, and vitamin D is due to a 9- to 10-month offset in solar irradiance which may change during gestation. Using the MOC, the starting point of embryonic development, and relating to ambient solar conditions is more fruitful in explaining the incidence of MS. However, significant MS genetic loading does obviously modify the environmental effects of UVR.

### 5.1 Regarding latitude, UVR, and CV_UVR_

From reviewing the above papers, latitude and UVR (particularly above 40 deg N or S latitude) are clearly more important in the pathogenesis of MS, followed by the VDR-vitamin D. Research reports that the incidence of MS is less in large cities compared to rural regions [43,80]. Tall buildings with abundant glass windows partially block UVR but approximately 30% of immunosuppressive UV-A penetrates most window glass while blocking 90% of genotoxic UV-B and its vitamin D-producing effect. Rural/farming regions may exceed the MS incidence of cities where there is more genotoxicity from unimpeded UV-B irradiance, primarily toxigenic in its effect. Chronic low-level UVR appears to be salutary for cardiovascular and some neoplastic diseases, but high-dose intermittent UVR predisposes to malignant melanoma particularly in areas with atmospheric ozone depletion as in Australia [81,82]. Note also that patients with MS tend to migrate to larger cities where there are more available social services, so MS prevalence there may be high, but MS incidence may be lower due to the factors mentioned above.

An important concept in this paper is the variation in UVR, e.g., CV_UVR_. Clues came from evidence that latitude is related to *variation* in suicide rates, and that rapid changes in sunlight at the autumnal equinox are associated with SAD. Variations in solar irradiance are associated with suicide attempts in bipolar 1 disorder [57]. High seasonal variation in sunlight was associated with bipolar disorder in an Arctic population again suggesting that variation in UVR is as important as intensity [83]. The rate of variation in UVR in the late summer and early fall is three times the rate in the spring, so persons conceived from July through September (born in April through June) are more likely to be predisposed to depression if there is associated genetic loading [84]. More variation in UVR in late summer and autumn was also found in Shenyang, China [85].

### 5.2 Regarding VDR

Vitamin D is considered a hormone because its ligand-dependent receptor, VDR, is ubiquitous in the body, including the brain, and regulates not only calcium metabolism, but also immunity, cell growth and differentiation, as well as energy metabolism [26,86]. It is intriguing that life on Earth has coopted energetic UVR by creating a receptor that controls so many biochemical reactions. We can look forward to new findings in this area of research.

### 5.3 Regarding race

The amount of melanin in human skin blocks UVR which may explain the increased incidence of MS in African Americans, especially women due to a decreased formation of vitamin D. In addition, the higher incidence of MS in women, especially over the past five decades, could be due to the availability of birth control methods, as well as voluntary termination of pregnancy not as readily available less than a century ago. In the past, women were destined to be pregnant for most of their reproductive years and the large number of offspring was an advantage in an agrarian economy. While pregnancy itself does not normally suppress the immune system, which is actually proinflammatory in the early stages, the placenta modulates immune reactions and post-partum breastfeeding after delivery appears to blunt autoimmune reactions that could lead to MS [87]. MS is just one of several autoimmune conditions seen more often in women. African Americans, aboriginally from +30 to −30° latitude, may be genetically adapted to less variable UVR, so migrating to higher latitudes exposes them to a greater CV_UVR_ and to more MS particularly in women for the reasons mentioned above.

### 5.4 Regarding geographic elevation

High spikes of UVR from more abundant sunspots can occur at any time in a solar cycle. With increasing altitude, UVR penetration increases due to less absorption in a thinning atmosphere. For example, In Denver, CO at 5,279 ft in elevation, UVR would increase approximately 4%/1000 feet or (5.3 × 4%) = approximately 21%. UVR doubles in intensity above the atmosphere during the approximately three years of a Solar MAX, but we measured only about a 13% increase in UVR in Cycle 24 MAX at ground level. Still, that increase is significant as UVR is genotoxic and mutagenic to DNA.

Solar Cycle 24 was one of our weaker cycles, the strongest being Cycle 19 from April 1954 and ending in October 1964, the MAX (1958–1961) being the strongest in 250 years. During that MAX, there was double the UVR compared to Cycle 24 and triggered more MMI during that period [33].

Of nine states with the highest MS incidence: Nebraska (41.5 deg, elevation 2,600 ft); Wyoming (43.1 deg, elevation 6,000 ft); Montana (46.9 deg, elevation 3,400 ft); Iowa (41.9 deg, 1,100 ft), Maine (45.3 deg, 600 ft), West Virginia (38.6 deg, 1,654 ft), Pennsylvania (41.2 deg, 1,100 ft); North Dakota (47.6 deg, 1,900 ft); South Dakota (44.0 deg, 2,200 ft); (Alaska (64.2 deg)- not used because most persons are immigrants), the average latitude is 44.2 deg N. The average elevation of the U. S. 50 states is 1,760 ft. The average elevation of the nine highest MS incidence states is 2,284 ft, 30% higher than all the states. The conclusion is that higher elevation does not protect against MS even with the presumed increased vitamin D production and promotes MS due to genotoxicity from the stronger UVR at higher elevation. In a previous study we [WEL & GED] found that the 13% increased UVR in six U.S. states with the highest elevations reduced average lifespan (of the general population, not MS patients) by about 3 months [88]. Note that large cities, like Denver, will concentrate MS patients from the state's rural areas primarily because of the support services located there.

That UVR is immunosuppressive for MS but being proinflammatory at higher elevations supports an irradiance threshold that overwhelms cellular DNA repair mechanisms. In our previous work [GED & WEL] we found that genotoxicity/mutagenicity occurred in 11% of days when SSN was greater than or equal to 90 SSN [71]. This finding is in the range of what we found in this paper, namely, that about 13% more energy than average is all that is required to produce genotoxicity. However, the critical elevation can be modified by poverty, poor diet, and variation in the genes related to MS.

### 5.5 Regarding MOC for other diseases (refer to Fig. 2)

**Fig. 2:**
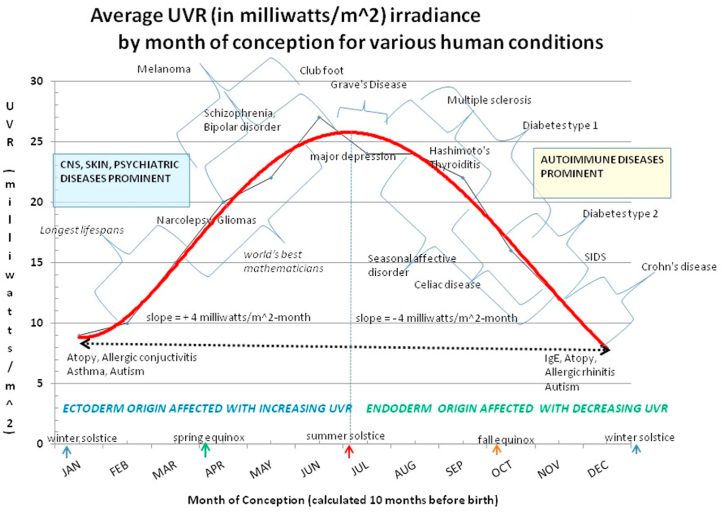
Diseases predisposed to certain Months-of-Birth arranged by Months-of-Conception.

We [GED & WEL] recently reported a table of a variety of diseases that many authors associated with certain months of birth [33]. The diseases in that table were converted to MOC and these were plotted here in Fig. 2. Note that diseases of the embryonic ectoderm, e. g., psychiatric, dermatologic and CNS diseases occur in MOC when UVR is increasing whereas decreasing UVR at MOC predisposes to diseases of embryonic endoderm, e. g., gut and 70–80% of the immune system as seen in Fig. 2. We hypothesize that increased UVR energy for maternal exposure affects the embryo not only as more available energy, but also as a potential external threat that would require more connection with the environment as expressed in the CNS and integument. We did not see the pattern in Fig. 2 with a MOB distribution. As an aside, we included the world's historically most famous mathematicians in the ascending portion of Fig. 2 and found that they were more often conceived in April, May, June, and July and therefore were born in January, February, March, and April. A repository of mathematician data reports that the best mathematicians were born in the first four months of the year and their CNS was presumably stimulated to be particularly clever in detecting patterns in our universe [89]. One might also question why diabetes type 2 was seen in the descending region of Fig. 2 as the disease is known to have a strong genetic component and might not be as susceptible to environmental/epigenetic effects. However, a recent report linked Covid-19 infection with precipitating diabetes type 2 through a presumed inflammatory, immune mechanism [90]. We believe Fig. 2 supports our hypothesis that early-life events at conception are important in triggering MS.

### 5.6 Regarding psychiatric disease

Our previous publications have shown an effect of UVR on MMI, and other publications have emphasized the effect of variable sunlight on depression. These observations were the stimulus for this study on MS, a disease clearly related to UVR and geographic latitude.

### 5.7 Regarding the maternal role in affecting the embryo

Later in adult life UVR exposure mitigates the relapsing of MS presumably by affecting Treg lymphocytes directly through the skin in addition to the production of vitamin D [91]. UV-B in excess is clearly damaging to the epidermis having, on average, nearly 20% more photonic energy than UV-A. UV-A can directly suppress the immune system as with phototherapy for psoriasis, e.g., PUVA or for atopic dermatitis, e.g., UVA-1 (340–400 nm) [91] UV-A, albeit weaker in its effect on cellular chromophores, produces reactive oxygen species (ROS) and constitutes 95% of UVR, but its biologic effects are not completely known. The characterization of the many effects of UVR is beyond the scope of this paper but it is increasingly clear that the skin is a peripheral neuroendocrine organ [92]. By some mechanism the human embryo receives maternal cues regarding the external electromagnetic environment through the immune system/placenta that modify primordial ectodermal and endodermal tissues. Characterizing that mechanism is important future research.

## 6 Conclusions

• UVR is the more important driver affecting the incidence of MS than vitamin D *per se.*
• Using MOC explains more of the dynamics in triggering MS than MOB.
• A high UVR exposure at conception may trigger MS by suppressing the VDRs, fixing their activity in later fetal development, and requiring more UVR in adulthood to suppress the immune system.
• A rapidly increasing CV_UVR_ from August to December may repress the expression of VDR.
• The vitamin D-VDR mechanism serves to mitigate the expression of MS, but if UVR is particularly intense, that mechanism may be overwhelmed at high geographic elevations (above approximately 3,000 feet) and at high UVR occurring at solar cycle maxima (about 3 years out of the average 11-year solar cycle) where UVR can become genotoxic resulting in more, not less MS.
• Maternal immune cells, like Treg lymphocytes, influence the embryonic response to UVR; increasing UVR favors the ectodermal anlage (CNS, cutaneous and psychiatric diseases), while decreasing UVR favors the endodermal anlage (autoimmune diseases of lung, gut, and endocrine system).
• Environmental effects of UVR may trigger the genetic elements of MS but only if the latter are present.

VI: LIMITATIONS OF THE STUDY: One site in central Maine, USA at latitude 44° N. The authors encourage replication of this study in other venues. The works of Carsten Carlberg and D. W. Eyles are excellent references detailing the distribution of VDRs; they state these receptors are in virtually all cells of the body, including the brain. Although extremely important, we believe the distribution of VDRs is outside the scope of our paper.

## Advantages of the study

Fifteen years of ground-level UVR data.

## Future work

We would like to acquire MS decedent data from the National Center for Health Statistics (NCHS) to calculate the proportion of patients conceived/born during the MAX of other solar cycles (19 through 22 or greater if available) to compare to the genotoxic effects of the strongest solar cycle in 250 years, the 19th cycle.

## Ethics approval and consent to participate

No data required consent and there were no identifiers. We required no specific human or animal subjects.

## Supplement

From NOAA showing sunspot numbers by month and year. https://www.ngdc.noaa.gov/stp/space-weather/solar-data/solar-indices/sunspot-numbers/american/lists/list_aavso-arssn_monthly.txt

## Production note

### Author contribution statement

George E. Davis, Jr.: Conceived and designed the experiments; Contributed reagents, materials, analysis tools or data; Wrote the paper.

Matthew J. Davis, Walter E. Lowell: Analyzed and interpreted the data; Wrote the paper.

### Data availability statement

Data will be made available on request.

## Declaration of competing interest

The authors declare that they have no known competing financial interests or personal relationships that could have appeared to influence the work reported in this paper.
