# Supplementary material for: Triggering multiple sclerosis at conception and early gestation: The variation in ultraviolet radiation is as important as its intensity
Source: Heliyon. 2023 Jun 3;9(6):e16954. doi: 10.1016/j.heliyon.2023.e16954 (PMC10279836; doi:10.1016/j.heliyon.2023.e16954)
Supplement: Multimedia component 1 [file mmc1.pdf]

YOB	MOB	SSN
1900	1	9.4
1900	2	13.6
1900	3	8.6
1900	4	16
1900	5	15.2
1900	6	12.1
1900	7	8.3
1900	8	4.3
1900	9	8.3
1900	10	12.9
1900	11	4.5
1900	12	0.3
1901	1	0.2
1901	2	2.4
1901	3	4.5
1901	4	0
1901	5	10.2
1901	6	5.8
1901	7	0.7
1901	8	1
1901	9	0.6
1901	10	3.7
1901	11	3.8
1901	12	0
1902	1	5.5
1902	2	0
1902	3	12.4
1902	4	0
1902	5	2.8
1902	6	1.4
1902	7	0.9
1902	8	2.3
1902	9	7.6
1902	10	16.3
1902	11	10.3
1902	12	1.1
1903	1	8.3
1903	2	17
1903	3	13.5
1903	4	26.1
1903	5	14.6
1903	6	16.3
1903	7	27.9
1903	8	28.8
1903	9	11.1
1903	10	38.9

YOB	MOB	SSN
1903	11	44.5
1903	12	45.6
1904	1	31.6
1904	2	24.5
1904	3	37.2
1904	4	43
1904	5	39.5
1904	6	41.9
1904	7	50.6
1904	8	58.2
1904	9	30.1
1904	10	54.2
1904	11	38
1904	12	54.6
1905	1	54.8
1905	2	85.8
1905	3	56.5
1905	4	39.3
1905	5	48
1905	6	49
1905	7	73
1905	8	58.8
1905	9	55
1905	10	78.7
1905	11	107.2
1905	12	55.5
1906	1	45.5
1906	2	31.3
1906	3	64.5
1906	4	55.3
1906	5	57.7
1906	6	63.2
1906	7	103.6
1906	8	47.7
1906	9	56.1
1906	10	17.8
1906	11	38.9
1906	12	64.7
1907	1	76.4
1907	2	108.2
1907	3	60.7
1907	4	52.6
1907	5	42.9
1907	6	40.4
1907	7	49.7
1907	8	54.3

1907	9	85
1907	10	65.4
1907	11	61.5
1907	12	47.3
1908	1	39.2
1908	2	33.9
1908	3	28.7
1908	4	57.6
1908	5	40.8
1908	6	48.1
1908	7	39.5
1908	8	90.5
1908	9	86.9
1908	10	32.3
1908	11	45.5
1908	12	39.5
1909	1	56.7
1909	2	46.6
1909	3	66.3
1909	4	32.3
1909	5	36
1909	6	22.6
1909	7	35.8
1909	8	23.1
1909	9	38.8
1909	10	58.4
1909	11	55.8
1909	12	54.2
1910	1	26.4
1910	2	31.5
1910	3	21.4
1910	4	8.4
1910	5	22.2
1910	6	12.3
1910	7	14.1
1910	8	11.5
1910	9	26.2
1910	10	38.3
1910	11	4.9
1910	12	5.8
1911	1	3.4
1911	2	9
1911	3	7.8
1911	4	16.5
1911	5	9
1911	6	2.2
1911	7	3.5
1911	8	4
1911	9	4
1911	10	2.6
1911	11	4.2
1911	12	2.2
1912	1	0.3
1912	2	0
1912	3	4.9
1912	4	4.5
1912	5	4.4
1912	6	4.1
1912	7	3
1912	8	0.3
1912	9	9.5
1912	10	4.6
1912	11	1.1
1912	12	6.4
1913	1	2.3
1913	2	2.9
1913	3	0.5
1913	4	0.9
1913	5	0
1913	6	0
1913	7	1.7
1913	8	0.2
1913	9	1.2
1913	10	3.1
1913	11	0.7
1913	12	3.8
1914	1	2.8
1914	2	2.6
1914	3	3.1
1914	4	17.3
1914	5	5.2
1914	6	11.4
1914	7	5.4
1914	8	7.7
1914	9	12.7
1914	10	8.2
1914	11	16.4
1914	12	22.3
1915	1	23
1915	2	42.3
1915	3	38.8
1915	4	41.3
1915	5	33
1915	6	68.8

1915	7	71.6
1915	8	69.6
1915	9	49.5
1915	10	53.5
1915	11	42.5
1915	12	34.5
1916	1	45.3
1916	2	55.4
1916	3	67
1916	4	71.8
1916	5	74.5
1916	6	67.7
1916	7	53.5
1916	8	35.2
1916	9	45.1
1916	10	50.7
1916	11	65.6
1916	12	53
1917	1	74.7
1917	2	71.9
1917	3	94.8
1917	4	74.7
1917	5	114.1
1917	6	114.9
1917	7	119.8
1917	8	154.5
1917	9	129.4
1917	10	72.2
1917	11	96.4
1917	12	129.3
1918	1	96
1918	2	65.3
1918	3	72.2
1918	4	80.5
1918	5	76.7
1918	6	59.4
1918	7	107.6
1918	8	101.7
1918	9	79.9
1918	10	85
1918	11	83.4
1918	12	59.2
1919	1	48.1
1919	2	79.5
1919	3	66.5
1919	4	51.8
1919	5	88.1
1919	6	111.2
1919	7	64.7
1919	8	69
1919	9	54.7
1919	10	52.8
1919	11	42
1919	12	34.9
1920	1	51.1
1920	2	53.9
1920	3	70.2
1920	4	14.8
1920	5	33.3
1920	6	38.7
1920	7	27.5
1920	8	19.2
1920	9	36.3
1920	10	49.6
1920	11	27.2
1920	12	29.9
1921	1	31.5
1921	2	28.3
1921	3	26.7
1921	4	32.4
1921	5	22.2
1921	6	33.7
1921	7	41.9
1921	8	22.8
1921	9	17.8
1921	10	18.2
1921	11	17.8
1921	12	20.3
1922	1	11.8
1922	2	26.4
1922	3	54.7
1922	4	11
1922	5	8
1922	6	5.8
1922	7	10.9
1922	8	6.5
1922	9	4.7
1922	10	6.2
1922	11	7.4
1922	12	17.5
1923	1	4.5
1923	2	1.5
1923	3	3.3
1923	4	6.1

1923	5	3.2
1923	6	9.1
1923	7	3.5
1923	8	0.5
1923	9	13.2
1923	10	11.6
1923	11	10
1923	12	2.8
1924	1	0.5
1924	2	5.1
1924	3	1.8
1924	4	11.3
1924	5	20.8
1924	6	24
1924	7	28.1
1924	8	19.3
1924	9	25.1
1924	10	25.6
1924	11	22.5
1924	12	16.5
1925	1	5.5
1925	2	23.2
1925	3	18
1925	4	31.7
1925	5	42.8
1925	6	47.5
1925	7	38.5
1925	8	37.9
1925	9	60.2
1925	10	69.2
1925	11	58.6
1925	12	98.6
1926	1	71.8
1926	2	69.9
1926	3	62.5
1926	4	38.5
1926	5	64.3
1926	6	73.5
1926	7	52.3
1926	8	61.6
1926	9	60.8
1926	10	71.5
1926	11	60.5
1926	12	79.4
1927	1	81.6
1927	2	93
1927	3	69.6
1927	4	93.5
1927	5	79.1
1927	6	59.1
1927	7	54.9
1927	8	53.8
1927	9	68.4
1927	10	63.1
1927	11	67.2
1927	12	45.2
1928	1	83.5
1928	2	73.5
1928	3	85.4
1928	4	80.6
1928	5	77
1928	6	91.4
1928	7	98
1928	8	83.8
1928	9	89.7
1928	10	61.4
1928	11	50.3
1928	12	59
1929	1	68.9
1929	2	62.8
1929	3	50.2
1929	4	52.8
1929	5	58.2
1929	6	71.9
1929	7	70.2
1929	8	65.8
1929	9	34.4
1929	10	54
1929	11	81.1
1929	12	108
1930	1	65.3
1930	2	49.9
1930	3	35
1930	4	38.2
1930	5	36.8
1930	6	28.8
1930	7	21.9
1930	8	24.9
1930	9	32.1
1930	10	34.4
1930	11	35.6
1930	12	25.8
1931	1	14.6
1931	2	43.1

1931	3	30	1935	2	20.5
1931	4	31.2	1935	3	23.1
1931	5	24.6	1935	4	12.2
1931	6	15.3	1935	5	27.3
1931	7	17.4	1935	6	45.7
1931	8	13	1935	7	33.9
1931	9	19	1935	8	30.1
1931	10	10	1935	9	42.1
1931	11	18.7	1935	10	53.2
1931	12	17.8	1935	11	64.2
1932	1	12.1	1935	12	61.5
1932	2	10.6	1936	1	62.8
1932	3	11.2	1936	2	74.3
1932	4	11.2	1936	3	77.1
1932	5	17.9	1936	4	74.9
1932	6	22.2	1936	5	54.6
1932	7	9.6	1936	6	70
1932	8	6.8	1936	7	52.3
1932	9	4	1936	8	87
1932	10	8.9	1936	9	76
1932	11	8.2	1936	10	89
1932	12	11	1936	11	115.4
1933	1	12.3	1936	12	123.4
1933	2	22.2	1937	1	132.5
1933	3	10.1	1937	2	128.5
1933	4	2.9	1937	3	83.9
1933	5	3.2	1937	4	109.3
1933	6	5.2	1937	5	116.7
1933	7	2.8	1937	6	130.3
1933	8	0.2	1937	7	145.1
1933	9	5.1	1937	8	137.7
1933	10	3	1937	9	100.7
1933	11	0.6	1937	10	124.9
1933	12	0.3	1937	11	74.4
1934	1	3.4	1937	12	88.8
1934	2	7.8	1938	1	98.4
1934	3	4.3	1938	2	119.2
1934	4	11.3	1938	3	86.5
1934	5	19.7	1938	4	101
1934	6	6.7	1938	5	127.4
1934	7	9.3	1938	6	97.5
1934	8	8.3	1938	7	165.3
1934	9	4	1938	8	115.7
1934	10	5.7	1938	9	89.6
1934	11	8.7	1938	10	99.1
1934	12	15.4	1938	11	122.2
1935	1	18.6	1938	12	92.7

1939	1	80.3
1939	2	77.4
1939	3	64.6
1939	4	109.1
1939	5	118.3
1939	6	101
1939	7	97.6
1939	8	105.8
1939	9	112.6
1939	10	88.1
1939	11	68.1
1939	12	42.1
1940	1	50.5
1940	2	59.4
1940	3	83.3
1940	4	60.7
1940	5	54.4
1940	6	83.9
1940	7	67.5
1940	8	105.5
1940	9	66.5
1940	10	55
1940	11	58.4
1940	12	68.3
1941	1	45.6
1941	2	44.5
1941	3	46.4
1941	4	32.8
1941	5	29.5
1941	6	59.8
1941	7	66.9
1941	8	60
1941	9	65.9
1941	10	46.3
1941	11	38.4
1941	12	33.7
1942	1	35.6
1942	2	52.8
1942	3	54.2
1942	4	60.7
1942	5	25
1942	6	11.4
1942	7	17.7
1942	8	20.2
1942	9	17.2
1942	10	19.2
1942	11	30.7
1942	12	22.5
1943	1	12.4
1943	2	28.9
1943	3	27.4
1943	4	26.1
1943	5	14.1
1943	6	7.6
1943	7	13.2
1943	8	19.4
1943	9	10
1943	10	7.8
1943	11	10.2
1943	12	18.8
1944	1	3.7
1944	2	0.5
1944	3	11
1944	4	0.3
1944	5	2.5
1944	6	5
1944	7	5
1944	8	16.7
1944	9	14.3
1944	10	16.9
1944	11	10.8
1944	12	28.4
1945	1	18.5
1945	2	12.7
1945	3	21.5
1945	4	32
1945	5	30.6
1945	6	36.2
1945	7	42.6
1945	8	25.9
1945	9	34.9
1945	10	68.8
1945	11	46
1945	12	27.4
1946	1	47.6
1946	2	86.2
1946	3	76.6
1946	4	75.7
1946	5	84.9
1946	6	73.5
1946	7	116.2
1946	8	107.2
1946	9	94.4
1946	10	102.3

1946	11	123.8
1946	12	121.7
1947	1	115.7
1947	2	133.4
1947	3	129.8
1947	4	149.8
1947	5	201.3
1947	6	163.9
1947	7	157.9
1947	8	188.8
1947	9	169.4
1947	10	163.6
1947	11	128
1947	12	116.5
1948	1	108.5
1948	2	86.1
1948	3	94.8
1948	4	189.7
1948	5	174
1948	6	167.8
1948	7	142.2
1948	8	157.9
1948	9	143.3
1948	10	136.3
1948	11	95.8
1948	12	138
1949	1	119.1
1949	2	182.3
1949	3	157.5
1949	4	147
1949	5	106.2
1949	6	121.7
1949	7	125.8
1949	8	123.8
1949	9	145.3
1949	10	131.6
1949	11	143.5
1949	12	117.6
1950	1	101.6
1950	2	94.8
1950	3	109.7
1950	4	113.4
1950	5	106.2
1950	6	83.6
1950	7	91
1950	8	85.2
1950	9	51.3
1950	10	61.4
1950	11	54.8
1950	12	54.1
1951	1	59.9
1951	2	59.9
1951	3	55.9
1951	4	92.9
1951	5	108.5
1951	6	100.6
1951	7	61.5
1951	8	61
1951	9	83.1
1951	10	51.6
1951	11	52.4
1951	12	45.8
1952	1	40.7
1952	2	22.7
1952	3	22
1952	4	29.1
1952	5	23.4
1952	6	36.4
1952	7	39.3
1952	8	54.9
1952	9	28.2
1952	10	23.8
1952	11	22.1
1952	12	34.3
1953	1	26.5
1953	2	3.9
1953	3	10
1953	4	27.8
1953	5	12.5
1953	6	21.8
1953	7	8.6
1953	8	23.5
1953	9	19.3
1953	10	8.2
1953	11	1.6
1953	12	2.5
1954	1	0.2
1954	2	0.5
1954	3	10.9
1954	4	1.8
1954	5	0.8
1954	6	0.2
1954	7	4.8
1954	8	8.4

1954	9	1.5
1954	10	7
1954	11	9.2
1954	12	7.6
1955	1	23.1
1955	2	20.8
1955	3	4.9
1955	4	11.3
1955	5	28.9
1955	6	31.7
1955	7	26.7
1955	8	40.7
1955	9	42.7
1955	10	58.5
1955	11	89.2
1955	12	76.9
1956	1	73.6
1956	2	124
1956	3	118.4
1956	4	110.7
1956	5	136.6
1956	6	116.6
1956	7	129.1
1956	8	169.6
1956	9	173.2
1956	10	155.3
1956	11	201.3
1956	12	192.1
1957	1	165
1957	2	130.2
1957	3	157.4
1957	4	175.2
1957	5	164.6
1957	6	200.7
1957	7	187.2
1957	8	158
1957	9	235.8
1957	10	253.8
1957	11	210.9
1957	12	239.4
1958	1	202.5
1958	2	164.9
1958	3	190.7
1958	4	196
1958	5	175.3
1958	6	171.5
1958	7	191.4
1958	8	200.2
1958	9	201.2
1958	10	181.5
1958	11	152.3
1958	12	187.6
1959	1	217.4
1959	2	143.1
1959	3	185.7
1959	4	163.3
1959	5	172
1959	6	168.7
1959	7	149.6
1959	8	199.6
1959	9	145.2
1959	10	111.4
1959	11	124
1959	12	125
1960	1	146.3
1960	2	106
1960	3	102.2
1960	4	122
1960	5	119.6
1960	6	110.2
1960	7	121.7
1960	8	134.1
1960	9	127.2
1960	10	82.8
1960	11	89.6
1960	12	85.6
1961	1	57.9
1961	2	46.1
1961	3	53
1961	4	61.4
1961	5	51
1961	6	77.4
1961	7	70.2
1961	8	55.8
1961	9	63.6
1961	10	37.7
1961	11	32.6
1961	12	39.9
1962	1	38.7
1962	2	50.3
1962	3	45.6
1962	4	46.4
1962	5	43.7
1962	6	42

1962	7	21.8
1962	8	21.8
1962	9	51.3
1962	10	39.5
1962	11	26.9
1962	12	23.2
1963	1	19.8
1963	2	24.4
1963	3	17.1
1963	4	29.3
1963	5	43
1963	6	35.9
1963	7	19.6
1963	8	33.2
1963	9	38.8
1963	10	35.3
1963	11	23.4
1963	12	14.9
1964	1	15.3
1964	2	17.7
1964	3	16.5
1964	4	8.6
1964	5	9.5
1964	6	9.1
1964	7	3.1
1964	8	9.3
1964	9	4.7
1964	10	6.1
1964	11	7.4
1964	12	15.1
1965	1	17.5
1965	2	14.2
1965	3	11.7
1965	4	6.8
1965	5	24.1
1965	6	15.9
1965	7	11.9
1965	8	8.9
1965	9	16.8
1965	10	20.1
1965	11	15.8
1965	12	17
1966	1	28.2
1966	2	24.4
1966	3	25.3
1966	4	48.7
1966	5	45.3

1966	6	47.7
1966	7	56.7
1966	8	51.2
1966	9	50.2
1966	10	57.2
1966	11	57.2
1966	12	70.4
1967	1	110.9
1967	2	93.6
1967	3	111.8
1967	4	69.5
1967	5	86.5
1967	6	67.3
1967	7	91.5
1967	8	107.2
1967	9	76.8
1967	10	88.2
1967	11	94.3
1967	12	126.4
1968	1	121.8
1968	2	111.9
1968	3	92.2
1968	4	81.2
1968	5	127.2
1968	6	110.3
1968	7	96.1
1968	8	109.3
1968	9	117.2
1968	10	107.7
1968	11	86
1968	12	109.8
1969	1	104.4
1969	2	120.5
1969	3	135.8
1969	4	106.8
1969	5	120
1969	6	106
1969	7	96.8
1969	8	98
1969	9	91.3
1969	10	95.7
1969	11	93.5
1969	12	97.9
1970	1	111.5
1970	2	127.8
1970	3	102.9
1970	4	109.5

1970	5	127.5
1970	6	106.8
1970	7	112.5
1970	8	93
1970	9	99.5
1970	10	86.6
1970	11	95.2
1970	12	83.5
1971	1	91.3
1971	2	79
1971	3	60.7
1971	4	71.8
1971	5	57.5
1971	6	49.8
1971	7	81
1971	8	61.4
1971	9	50.2
1971	10	51.7
1971	11	63.2
1971	12	82.2
1972	1	61.5
1972	2	88.4
1972	3	80.1
1972	4	63.2
1972	5	80.5
1972	6	88
1972	7	76.5
1972	8	76.8
1972	9	64
1972	10	61.3
1972	11	41.6
1972	12	45.3
1973	1	43.4
1973	2	42.9
1973	3	46
1973	4	57.7
1973	5	42.4
1973	6	37.5
1973	7	23.1
1973	8	25.6
1973	9	59.3
1973	10	30.7
1973	11	23.9
1973	12	23.3
1974	1	27.6
1974	2	26
1974	3	21.3
1974	4	40.3
1974	5	39.5
1974	6	36
1974	7	55.8
1974	8	33.6
1974	9	40.2
1974	10	47.1
1974	11	25
1974	12	20.5
1975	1	18.9
1975	2	11.5
1975	3	11.5
1975	4	5.1
1975	5	9
1975	6	11.4
1975	7	28.2
1975	8	39.7
1975	9	13.9
1975	10	9.1
1975	11	19.4
1975	12	7.8
1976	1	8.1
1976	2	4.3
1976	3	21.9
1976	4	18.8
1976	5	12.4
1976	6	12.2
1976	7	1.9
1976	8	16.4
1976	9	13.5
1976	10	20.6
1976	11	5.2
1976	12	15.3
1977	1	16.4
1977	2	23.1
1977	3	8.7
1977	4	12.9
1977	5	18.6
1977	6	38.5
1977	7	21.4
1977	8	30.1
1977	9	44
1977	10	43.8
1977	11	29.1
1977	12	43.2
1978	1	51.9
1978	2	93.6

1978	3	76.5
1978	4	99.7
1978	5	82.7
1978	6	95.1
1978	7	70.4
1978	8	58.1
1978	9	138.2
1978	10	125.1
1978	11	97.9
1978	12	122.7
1979	1	166.6
1979	2	137.5
1979	3	138
1979	4	101.5
1979	5	134.4
1979	6	149.5
1979	7	159.4
1979	8	142.2
1979	9	188.4
1979	10	186.2
1979	11	183.3
1979	12	176.3
1980	1	159.6
1980	2	155
1980	3	126.2
1980	4	164.1
1980	5	179.9
1980	6	157.3
1980	7	136.3
1980	8	135.4
1980	9	155
1980	10	164.7
1980	11	147.9
1980	12	174.4
1981	1	114
1981	2	141.3
1981	3	135.5
1981	4	156.4
1981	5	127.5
1981	6	90.9
1981	7	143.8
1981	8	158.7
1981	9	167.3
1981	10	162.4
1981	11	137.5
1981	12	150.1
1982	1	111.2
1982	2	163.6
1982	3	153.8
1982	4	122
1982	5	82.2
1982	6	110.4
1982	7	106.1
1982	8	107.6
1982	9	118.8
1982	10	94.7
1982	11	98.1
1982	12	127
1983	1	84.3
1983	2	51
1983	3	66.5
1983	4	80.7
1983	5	99.2
1983	6	91.1
1983	7	82.2
1983	8	71.8
1983	9	50.3
1983	10	55.8
1983	11	33.3
1983	12	33.4
1984	1	57
1984	2	85.4
1984	3	83.5
1984	4	69.7
1984	5	76.4
1984	6	46.1
1984	7	37.4
1984	8	25.5
1984	9	15.7
1984	10	12
1984	11	22.8
1984	12	18.7
1985	1	16.5
1985	2	15.9
1985	3	17.2
1985	4	16.2
1985	5	27.5
1985	6	24.2
1985	7	30.7
1985	8	11.1
1985	9	3.9
1985	10	18.6
1985	11	16.2
1985	12	17.3

1986	1	2.5
1986	2	23.2
1986	3	15.1
1986	4	18.5
1986	5	13.7
1986	6	1.1
1986	7	18.1
1986	8	7.4
1986	9	3.8
1986	10	35.4
1986	11	15.2
1986	12	6.8
1987	1	10.4
1987	2	2.4
1987	3	14.7
1987	4	39.6
1987	5	33
1987	6	17.4
1987	7	33
1987	8	38.7
1987	9	33.9
1987	10	60.6
1987	11	39.9
1987	12	27.1
1988	1	59
1988	2	40
1988	3	76.2
1988	4	88
1988	5	60.1
1988	6	101.8
1988	7	113.8
1988	8	111.6
1988	9	120.1
1988	10	125.1
1988	11	125.1
1988	12	179.2
1989	1	161.3
1989	2	165.1
1989	3	131.4
1989	4	130.6
1989	5	138.5
1989	6	196.2
1989	7	126.9
1989	8	168.9
1989	9	176.7
1989	10	159.4
1989	11	173
1989	12	165.5
1990	1	177.3
1990	2	130.5
1990	3	140.3
1990	4	140.3
1990	5	132.2
1990	6	105.4
1990	7	149.4
1990	8	200.3
1990	9	125.2
1990	10	145.5
1990	11	131.4
1990	12	129.7
1991	1	136.9
1991	2	167.5
1991	3	141.9
1991	4	140
1991	5	121.3
1991	6	169.7
1991	7	173.7
1991	8	176.3
1991	9	125.3
1991	10	144.1
1991	11	108.2
1991	12	144.4
1992	1	150
1992	2	161.1
1992	3	106.7
1992	4	99.8
1992	5	73.8
1992	6	65.2
1992	7	85.7
1992	8	64.5
1992	9	63.9
1992	10	88.7
1992	11	91.8
1992	12	82.6
1993	1	59.3
1993	2	91
1993	3	69.8
1993	4	62.2
1993	5	61.3
1993	6	49.8
1993	7	57.9
1993	8	42.2
1993	9	22.4
1993	10	56.4

1993	11	35.6
1993	12	48.9
1994	1	57.8
1994	2	35.5
1994	3	31.7
1994	4	16.1
1994	5	17.8
1994	6	28
1994	7	35.1
1994	8	22.5
1994	9	25.7
1994	10	44
1994	11	18
1994	12	26.2
1995	1	24.2
1995	2	29.9
1995	3	31.1
1995	4	14
1995	5	14.5
1995	6	15.6
1995	7	14.5
1995	8	14.3
1995	9	11.8
1995	10	21.1
1995	11	9
1995	12	10
1996	1	11.5
1996	2	4.4
1996	3	9.2
1996	4	4.8
1996	5	5.5
1996	6	11.8
1996	7	8.2
1996	8	14.4
1996	9	1.6
1996	10	0.9
1996	11	17.9
1996	12	13.3
1997	1	5.7
1997	2	7.6
1997	3	8.7
1997	4	15.5
1997	5	18.5
1997	6	12.7
1997	7	10.4
1997	8	24.4
1997	9	51.3
1997	10	23.8
1997	11	39
1997	12	41.2
1998	1	31.9
1998	2	40.3
1998	3	54.8
1998	4	53.4
1998	5	56.3
1998	6	70.7
1998	7	66.6
1998	8	92.2
1998	9	92.9
1998	10	55.5
1998	11	74
1998	12	81.9
1999	1	62
1999	2	66.3
1999	3	68.8
1999	4	63.7
1999	5	106.4
1999	6	137.7
1999	7	113.5
1999	8	93.7
1999	9	71.5
1999	10	116.7
1999	11	133.2
1999	12	84.6
2000	1	90.1
2000	2	112.9
2000	3	138.5
2000	4	125.5
2000	5	121.6
2000	6	124.9
2000	7	170.1
2000	8	130.5
2000	9	109.7
2000	10	99.4
2000	11	106.8
2000	12	104.4
2001	1	95.6
2001	2	80.6
2001	3	113.5
2001	4	107.7
2001	5	96.6
2001	6	134
2001	7	81.8
2001	8	106.4

2001	9	150.7
2001	10	125.5
2001	11	106.5
2001	12	132.2
2002	1	114.1
2002	2	107.4
2002	3	98.4
2002	4	120.7
2002	5	120.8
2002	6	88.3
2002	7	99.6
2002	8	116.4
2002	9	109.6
2002	10	97.5
2002	11	95.5
2002	12	80.8
2003	1	79.7
2003	2	46
2003	3	61.1
2003	4	60
2003	5	54.6
2003	6	77.4
2003	7	83.3
2003	8	72.7
2003	9	48.7
2003	10	65.5
2003	11	67.3
2003	12	46.5
2004	1	37.3
2004	2	45.8
2004	3	49.1
2004	4	39.3
2004	5	41.5
2004	6	43.2
2004	7	51.1
2004	8	40.9
2004	9	27.7
2004	10	48
2004	11	43.5
2004	12	17.9
2005	1	31.3
2005	2	29.2
2005	3	24.5
2005	4	24.2
2005	5	42.7
2005	6	39.3
2005	7	40.1
2005	8	36.4
2005	9	21.9
2005	10	8.7
2005	11	18
2005	12	41.1
2006	1	15.3
2006	2	4.9
2006	3	10.6
2006	4	30.2
2006	5	22.3
2006	6	13.9
2006	7	12.2
2006	8	12.9
2006	9	14.4
2006	10	10.5
2006	11	21.4
2006	12	13.6
2007	1	16.8
2007	2	10.7
2007	3	4.5
2007	4	3.4
2007	5	11.7
2007	6	12.1
2007	7	9.7
2007	8	6
2007	9	2.4
2007	10	0.9
2007	11	1.7
2007	12	10.1
2008	1	3.3
2008	2	2.1
2008	3	9.3
2008	4	2.9
2008	5	3.2
2008	6	3.4
2008	7	0.8
2008	8	0.5
2008	9	1.1
2008	10	2.9
2008	11	4.1
2008	12	0.8
2009	1	1.5
2009	2	1.4
